# Tackling the lack of diversity in cancer research

**DOI:** 10.1242/dmm.050275

**Published:** 2023-09-08

**Authors:** Christian Molina-Aguilar, C. Daniela Robles-Espinoza

**Affiliations:** ^1^ Laboratorio Internacional de Investigación sobre el Genoma Humano, Universidad Nacional Autónoma de México, Santiago de Querétaro 76230, Mexico; ^2^ Cancer, Ageing and Somatic Mutation, Wellcome Sanger Institute, Hinxton, Cambridgeshire CB10 1SA, UK

## Abstract

Despite the clear benefit of studying biological samples from diverse genetic backgrounds and geographical locations, our current knowledge of disease is mostly derived from the study of European-descent individuals. In the cancer field, this is reflected in the poor representation of African and Amerindian/Latino samples in most large public data repositories. This lack of diversity is due to several reasons, but here we focus on (1) the lack of support for studies on non-European populations that are performed in low- and middle-income countries (LMICs), and (2) unequal partnerships between scientists in LMICs and those in high-income countries. We argue that expanding access to research funding, increasing the participation of underrepresented scientists in editorial boards and international conferences, facilitating the publication of studies conducted in these countries, and properly acknowledging LMIC researchers' contributions in publications and grant applications will promote equity for scientists working in LMICs. We envisage that this will translate to more impactful research in these countries, which will include more samples from diverse populations. For the cancer field, this will broaden our understanding of pathomechanisms and may help to improve the treatment of patients from all backgrounds.

## Teaser

**Summary:** We believe that increasing opportunities for scientists in low- and middle-income countries will translate to more impactful research done in these countries that inherently includes more samples from diverse populations.

## Differences in cancer incidence and biomarkers across populations, and the importance of diversity for patient stratification

The study of biological samples from populations of diverse ethnicities and geographical locations is essential to achieve a more complete understanding of (patho)biological processes, as well as to tailor medical interventions for all patients. Cancer risk and prevalence can vary depending on genetic ancestry. For example, the 8q24 chromosomal region is enriched with risk variants for prostate cancer, which are substantially more common in individuals of African ancestry (Han et al., 2016; Walavalkar et al., 2020), and the *PLCE1* gene has been found to contain genetic variants proposed to explain, in part, the high rate of gastric cancer in the Chinese population (Abnet et al., 2010). Furthermore, nasopharyngeal cancer is particularly prevalent in East and Southeast Asia (Chen et al., 2019), and triple-negative breast cancer, which has worse prognosis than other breast cancer subtypes, represents a higher proportion of total breast cancer cases in individuals of African and Hispanic ancestry (Plasilova et al., 2016; Siddharth and Sharma, 2018; Zevallos et al., 2020). Similarly, some cancer types are more prevalent in certain subpopulations. For example, gallbladder cancer is disproportionally more common in Southern Chile, particularly in individuals of Mapuche ancestry, which is also associated with increased gallbladder cancer mortality (Andia et al., 2008; Lorenzo Bermejo et al., 2017; Olivares, 2016). There is also evidence to suggest that increasing the genetic ancestry diversity of reference panels can directly translate into better patient stratification. A recent, large study found that tumour mutational burden, a biomarker used to stratify patients for immune checkpoint inhibitor treatment, is more frequently overestimated in non-European than in European patients (Nassar et al., 2022). This means that patients of non-European ancestry are more frequently misclassified as potentially benefitting from these treatments. The same study also identified a potential cancer biomarker – alterations in expression of the *MGA* gene – with opposing prognostic effects in patients with differing ancestry, although the authors warn that small sample sizes may mean that their conclusions are not definitive. In general, differences in methylation and mRNA expression of hundreds of genes, as well as potential differences in immune-related features that may influence immunotherapy response, have been observed across ancestries (Carrot-Zhang et al., 2020). Additionally, it has been shown that polygenic risk scores derived from the genome-wide association studies of European samples do not have comparable performance in other populations (Duncan et al., 2019; Martin et al., 2017). Therefore, large studies with more diverse populations are needed to improve cancer diagnosis, patient stratification and, potentially, prognosis based on genetics.

As mentioned above, differences in cancer incidence can be due to genetic ancestry in certain populations, but cancer incidence is also impacted by environmental and, importantly, social factors that affect health. For example, differences in exposure to carcinogenic factors, such as viral infections and environmental pollution that correlate with socioeconomic status, explain some of the variability in cancer incidence seen across populations (Miller et al., 2018; Minas et al., 2020; Pham et al., 2018; Zavala et al., 2021). This illustrates that only by studying a large and diverse collection of individuals, living in different communities and countries, can we understand the myriad risk factors and mutational processes that drive cancer and how genetics and the environment interact to promote tumour growth. A deeper understanding of these factors will translate into improved screening, for example for cancer types more prevalent in certain ancestries and geographical locations, and better patient stratification for therapies.

[…] patients of non-European ancestry are more frequently misclassified as potentially benefitting from these [immune checkpoint inhibitor] treatments.

## Bias in cancer catalogues

**Fig. 1.**
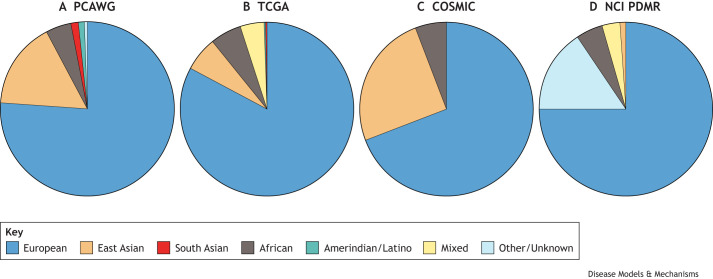
**There is a lack of representation of samples from non-European ancestry in large public repositories.** (A-D) The pie charts depict samples in each collection by ancestry in the PanCancer Analysis of Whole Genomes (PCAWG; A), The Cancer Genome Atlas (TCGA; B), the Catalogue of Somatic Mutations in Cancer (COSMIC) Cell Lines Project (C) and the National Cancer Institute (NCI) Patient-Derived Models Repository (PDMR) (D). (A) The PCAWG contains 2583 high-quality samples, including 1968 (76.2%) labelled as European, 416 (16.1%) labelled as East Asian, 124 (4.8%) labelled as African, 35 (1.4%) labelled as South Asian, 26 (1%) labelled as Admixed American/Amerindian/Latino and 14 (0.5%) labelled as Unknown (Campbell et al., 2020). (B) TCGA, as analysed by Carrot-Zhang et al. (2020), contains 10,678 samples, including 8836 (82.7%) labelled as European, 669 (6.3%) labelled as East Asian, 651 (6%) labelled as African, 41 (0.4%) labelled as Native/Latin American, 27 (0.3%) labelled as South Asian and 454 (4.3%) labelled as having at least 20% admixed descent. (C) The COSMIC Cell Lines Project contains 1007 samples, including 308 with ancestry labelled by COSMIC and 699 with ancestry labelled by Kessler et al. (2019), together including 697 (69.2%) labelled as European, 253 (25.1%) labelled as East Asian, 56 (5.6%) labelled as African and 1 (<0.1%) labelled as South Asian. (D) The NCI PDMR contains 979 samples, with 734 (75%) labell­ed as European, 155 (15.8%) labelled as ‘not applicable’, 49 (5%) labelled as West African, 31 (3.2%) labelled as Mixed (all ancestries, including Native and Latin American <80%), 9 (<1%) labelled as East Asian and 1 (<1%) labelled as Native/Latin American.

Currently, the clinical and genomic data in cancer catalogues are not representative of the world's ethnic and geographical diversity (Fig. 1). More than 80% of samples analysed by The Cancer Genome Atlas (TCGA), an international effort that aimed to characterise the genomic profile of more than 30 cancer types, were classified as being of European ancestry (Carrot-Zhang et al., 2020). Another example is the PanCancer Analysis of Whole Genomes (PCAWG), an international collaboration that profiled 2658 tumour samples by whole-genome sequencing and other large-scale analyses, which contains only ∼1% and 5% of samples with predominantly Amerindian and African ancestry, respectively (Campbell et al., 2020). Similarly, ∼70% of commonly used cell lines found in databases and repositories, such as the Catalogue of Somatic Mutations in Cancer (COSMIC) and the National Cancer Institute (NCI) Patient-Derived Models Repository (PDMR), are of European ancestry (Kessler et al., 2019; Raghavan, 2022). This diversity problem is, of course, not limited to this research area. For example, beyond cancer catalogues, the genome aggregation database, gnomAD, had only ∼8% and 12% of genome sequences classified as African and Latino, respectively (Karczewski et al., 2020). This lack of diversity in cohorts biases our knowledge of the aetiology and mechanisms of cancer, as well as of other diseases, which has an impact on patient stratification and treatment, as described above.

**Figure:**
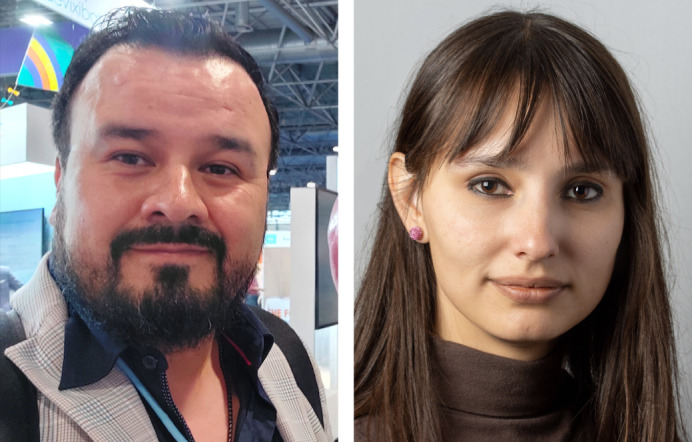
Christian Molina-Aguilar (left) and C. Daniela Robles-Espinoza (right)

## Persisting bias

The reasons why this lack of diversity in study cohorts persists are complex. Two major factors are a bias towards European ancestry in population and study cohort representation within high-income countries (HICs) (Bentley et al., 2017; Bycroft et al., 2018), and a lack of well-funded studies being conducted in low- and middle-income countries (LMICs) in which the majority of the population is of non-European descent. In this Perspective, we will focus on the latter. An analysis of the international GWAS Catalog found that, from 2009 to 2016, participants of non-European descent increased from 4% to 19%, with this being mostly due to an increase in Asian population studies conducted in Asian countries, and with African and Hispanic or Latin American ancestry representation increasing only modestly (Popejoy and Fullerton, 2016). We propose that representation of certain populations in databases could be lagging behind because most countries with predominantly African or Amerindian populations are categorised as LMICs, which invest modestly in research and development, typically less than 1% of the gross domestic product (UNESCO Institute for Statistics, 2023). This results in limited grant support (Ciocca and Delgado, 2017; Robles-Espinoza, 2021; World RePORT, 2023). Furthermore, as we are based in an LMIC (Box 1), we have experienced extra import taxes and long transportation waiting times for research materials, as have others (Ciocca and Delgado, 2017; Patel et al., 2016). The combination of these factors may result in fewer, smaller and more limited scientific studies.

**Box 1. Author information**

C.M.-A. is a postdoctoral researcher at the International Laboratory for Human Genome Research (LIIGH), part of the National Autonomous University of Mexico (UNAM). He studied his BSc and PhD in Mexico, and has spent the entirety of his research career in the country. He is mainly interested in the study of liver diseases from early to advanced stages, including hepatocellular carcinoma, using cell lines and animal models in order to have a better understanding of the genetic contribution to the development of these diseases. C.D.R.-E. is a group leader at LIIGH-UNAM. She studied her BSc in Mexico and her PhD in the UK, then returned to Mexico at the end of 2015 to start a research group. She is interested in studying the influence that genetics and the environment have on the somatic profile of tumours, mainly in the most common type of melanoma in Mexico and in chronic liver diseases.

Additionally, researchers based in LMICs also face barriers in science communication, and their studies often do not get the attention that those from well-funded institutes in HICs do. They generally have worse peer-review outcomes (Liu et al., 2023; Smith et al., 2023) and therefore tend to end up in journals with less visibility. This in turn may have an impact on researchers' career prospects (Moher et al., 2018), negatively influencing opportunities to get invited to conferences and access competitive grants, thus perpetuating the cycle. Publishing fees are also a large barrier, as, in many cases, the cost of publishing only one manuscript can amount up to several months of a researcher's salary (Peterson et al., 2013). On top of this, researchers in LMICs endure exclusion from access to published literature, not only due to steep journal subscription fees in many instances but also due to the language barrier. All of this is reflected in lower representation of LMIC researchers in international meetings, editorial and scientific society boards, and limited opportunities to attract international researchers to their laboratories. All of these factors impact the ability that researchers in LMICs have to undertake their studies, collaborate and publish. As these researchers would have greater access to samples from populations currently underrepresented in cancer research, this then impacts sample diversity in published datasets.

One way to channel more funding and resources to scientists in LMICs, and therefore enhance diversity in published datasets, is to form collaborations between researchers in LMICs and HICs that serve the interests of HICs and LMICs. However, these collaborations can take the form of unequal research partnerships between scientists in HICs and LMICs (Haelewaters et al., 2021; Rubagumya et al., 2022), without strengthening local cancer research capabilities. This phenomenon has been referred to as ‘helicopter research’. Many early publications studying diverse populations in LMICs were predominantly authored by teams from institutions in HICs and may not have meaningfully involved researchers working locally (Hoekman et al., 2012; Wong et al., 2014). A recent study found that ∼6.5% of cancer genomics studies using samples from African countries did not include any authors based in these countries, and nearly 15% and 17% of studies had first or last authors, respectively, based in countries outside Africa (Rotimi et al., 2021). We believe that this is a missed opportunity to enrich these studies with local perspectives and knowledge, and dismisses the experience and information that resident research communities have acquired over long periods of time. Moreover, clinical trials with exploitative practices have been run in LMICs, for example with substandard control arms – that is, comparing a new treatment against a suboptimal treatment instead of the standard-of-care treatment under the guise of local inaccessibility to the standard of care (Grover et al., 2016; Gyawali et al., 2022). Furthermore, cancer drug approval in LMICs that have hosted clinical trials is much slower than that in HICs, and, in many cases, these drugs are too expensive for patients in LMICs to access (Fundytus et al., 2021; Miller et al., 2021). We hypothesise that the persisting phenomenon of helicopter research can lead to distrust on the part of LMIC-based researchers, leading to fragile collaborations and fewer publications focusing on underrepresented populations within LMICs.

Where collaborations between scientists in HICs and LMICs are established, they should include capacity building, training opportunities for scientists in resource-poor locations, and a recognition of the value and knowledge that these researchers can bring to a project.

## What are the solutions?

So, what can be done to improve research in LMICs and, thus, the diversity of biological sample catalogues? Until governments in LMICs recognise the importance of expanding public expenditure in research, we believe that principal investigators (PIs) based in LMICs should be eligible for large research grants in HICs, preferably without the requirement that collaborators from HICs be necessarily involved. Many funding bodies already allow researchers from LMICs to submit grants as PIs. Notably, and in our experience, the Wellcome Trust, the Medical Research Council (MRC) and the Academy of Medical Sciences from the United Kingdom, the International Centre for Genetic Engineering and Biotechnology based in Italy, India and South Africa, and the Melanoma Research Alliance in the United States, all offer funding to PIs in LMICs. Furthermore, some larger grants, like the Applied Global Health Research grant from the MRC, and the Cancer grant from the Global Alliance for Chronic Diseases, explicitly encourage equitable partnerships between investigators in LMICs and HICs to provide benefits to all participants. These efforts are welcome and are undoubtedly helping to alleviate the limited research funding available within LMICs, but should be expanded. It is worth noting that nearly all these funders have policies that require grant holders to make data available in a suitable public repository; therefore, extra support may be required from the funders for LMIC researchers that do not have the resources to store this data.

In terms of helicopter research, there are signs that things are improving. The H3Africa initiative has facilitated or contributed to studies done in African populations that were coordinated by Africa-based researchers (Adebamowo et al., 2017; Ezeome et al., 2022; Sallah et al., 2017). As mentioned above, a recent study found that only ∼6.5% of cancer genomics studies using African biospecimens did not include any authors based in African institutions, although the study did not consider the evolution of authorship patterns through time (Rotimi et al., 2021). Furthermore, the proportion of LMIC-based authors in publications of oncology randomised controlled trials increased from 20% to 29% in the period from 1998 to 2008 (Wong et al., 2014). Where collaborations between scientists in HICs and LMICs are established, they should include capacity building, training opportunities for scientists in resource-poor locations, and a recognition of the value and knowledge that these researchers can bring to a project. Scientific projects should be planned with the participation of LMIC scientists from the beginning, and research agendas should be set considering the research priorities of all participants. We are convinced that these actions will improve research conditions in LMICs, establishing much-needed infrastructure and helping educate a highly skilled workforce, further supporting the study of local population samples.

Regarding scientific publishing, it has been shown that double-anonymised peer review can help close the gap in overall acceptance rates between authors from HICs and LMICs (Smith et al., 2023), so perhaps this policy should be more widely considered. Another support mechanism is heavy discounts or waivers on article processing fees for authors based in LMICs. Some institutions, such as the Electronic Information for Libraries (EIFL), can negotiate article processing charges for researchers in LMIC partner countries, and journals, such as Disease Models & Mechanisms, offer waivers on article processing fees to LMIC-based authors, or even authors based in HICs without sufficient funds. Initiatives that increase literature access can also help alleviate inequities between researchers in LMICs and HICs. For example, Plan S, in the United Kingdom, and SPARC, in the United States, are initiatives that require that all publicly funded research be published open access. We also welcome the opportunity to publish abstracts in the authors' local language, offered by the British Medical Journal. All of these opportunities will support local research environments, which we hypothesise will ultimately translate into an increase in high-quality studies stemming from LMICs.

We would also recommend working to increase the representation of scientists from LMICs in editorial boards (Melhem et al., 2022) and as invited speakers in international conferences. This would require better recognition of their expertise and perhaps broadening the definition of ‘cutting-edge’ research. For example, single-cell sequencing is currently considered cutting edge but is only feasible in a few highly resourced places, whereas epidemiological studies that do not require such specialised resources should be similarly respected as they can help understand disease behaviour and characteristics globally. These efforts should be spearheaded by scientists in HICs, who are typically in charge of organising large meetings and overseeing journal editorial boards. A good start would be to reach out to national specialised societies (for example, Asian, Latin American or African medical and/or biological societies) and make use of online science communities (e.g. The Science Forum, the Node or thematic Slack channels) to announce available positions and opportunities.

To support more LMIC-led research, many funders, journals and institutions have implemented formal recommendations and policies to maximise the utility and applicability of research to local communities, and to acknowledge the contributions of authors from LMICs in publications and grant applications (Table 1). One such set of guidelines has been collected in the Cape Town Statement, seeking to promote equity, diversity and fairness in research (Horn et al., 2023). This set of 20 recommendations includes guidelines for improving fair and equitable practices at all stages of research – from research proposal development and funding to data reporting – and proposes that HIC-based researchers should help alleviate the burden that data sharing mandates put on LMIC-based researchers, given the infrastructure and investment in data storage needed. In another example, the Royal Society of Chemistry has recently launched the ‘Joint commitment for action on inclusion and diversity in publishing’, which has brought together more than 50 publishing organisations (including The Company of Biologists, Elsevier, eLife, Science and others) seeking to improve diversity and inclusion by setting minimum standards and listing concrete actions (Odeny and Bosurgi, 2022). Nature Portfolio journals encourage authors to follow the recommendations of the ‘Global Code of Conduct for Research in Resource-Poor Settings’ (No author listed, 2022), which seeks to make sure that research is carried out ethically in LMICs. This considers the relevance of the proposed research for the LMIC partner, the inclusion of local researchers and community members, ethical access to samples, and other recommendations to ensure fairness, respect, care and honesty among collaborators. Wellcome has made efforts to diversify their funding committees and launched the Equality, Diversity and Inclusion in Science and Health Research initiative to tackle these inequalities. We hypothesise that these and other similar actions will have a noticeable impact in the formation of equitable and fair research teams, and will help set up an appropriate environment for recruitment of underrepresented patients and data sharing.

**Table 1.**
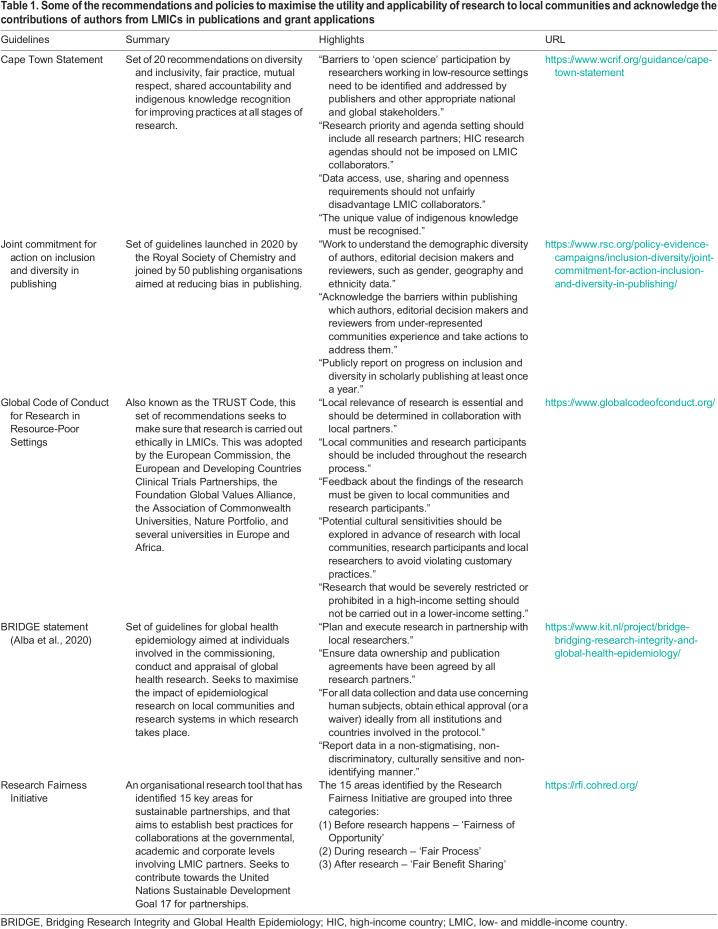
Some of the recommendations and policies to maximise the utility and applicability of research to local communities and acknowledge the contributions of authors from LMICs in publications and grant applications

In summary, we hypothesise that supporting LMIC-based researchers will increase the contribution that LMICs make to research outputs, which will, in turn, increase the study of samples from these regions and thus the diversity of data hosted in large repositories. There are, of course, other issues that contribute to the lack of diversity in cancer repositories, including cohort recruitment issues within HICs. Therefore, any actions taken must also consider these broader issues in scientific research that are driven by deeply ingrained societal barriers. Furthermore, many of the recommendations discussed above would also benefit researchers within ‘less prestigious’ research institutes in HICs, such as double-anonymised peer review. By supporting global cancer research efforts that expand the diversity of study populations, we will be able to unpick genetic and environmental risk factors in cancer and enhance therapy development and stratification. We hope that, in this way, we will ensure that our biological knowledge of cancer is derived from, and applicable to, people from all over the world.
